# Daratumumab: Dawn of a New Paradigm in Multiple Myeloma?

**DOI:** 10.6004/jadpro.2017.8.1.7

**Published:** 2017-01-01

**Authors:** Joseph A. Kalis

**Affiliations:** UCHealth Memorial Hospital, Colorado Springs, Colorado

Multiple myeloma is a neoplasm of the terminally differentiated B-lymphocyte, or plasma cell (Rollig, Knop, & Bornhauser, 2015). The second most common hematologic malignancy in the United States, multiple myeloma accounts for approximately 18% of all hematologic malignancies and 2% of all newly diagnosed cancers. The American Cancer Society estimated 30,330 new cases of multiple myeloma in the United States in 2016, with an estimated 12,430 deaths (Siegel, Miller, & Jamal, 2016). Although the rate of new multiple myeloma diagnoses is rising an average of 0.8% each year, the 5-year survival rate increased from 27% in 1975 to 47% in 2011 (Surveillance, Epidemiology, and End Results [SEER] Program, 2016).

While current therapies offer many patients with myeloma the prospect of long-term disease control, treatment responses are ultimately transient and relapse is inevitable. Even with the advent of drugs initially used for refractory disease, such as pomalidomide (Pomalyst) and carfilzomib (Kyprolis), resistance develops swiftly, and progression-free survival (PFS) remains brief (Siegel et al., 2012; San Miguel et al., 2013). Patients with myeloma refractory to proteasome inhibitors (PIs) and immunomodulatory drugs (IMiDs) have a median overall survival (OS) of 9 months, underscoring the need for fresh agents and novel mechanisms of action (Kumar et al., 2012).

As understanding of the bone marrow and myeloma microenvironments has increased, so too has the array of potential drug targets (Anderson, 2011; Mimura, Hideshima, & Anderson, 2015). A promising therapeutic avenue in myeloma is the use of monoclonal antibodies, as this class of drug offers new mechanisms of action and exhibits few off-target effects.

CD38 is a transmembrane glycoprotein regulating cell adhesion, cytoplasmic calcium flux, and mediation of signal transduction. Expressed by lymphoid and myeloid cells alike, CD38 is found on precursor and activated B and T cells, natural killer (NK) cells, erythrocytes, platelets, and plasma cells (Deaglio et al., 2007; Malavasi et al., 2008). CD38 is uniformly overexpressed in all stages of myeloma, including on myeloma plasma cell precursors and possibly myeloma stem cells. Additionally, CD38 is expressed at relatively low levels on normal lymphoid and myeloid cells, making it an attractive candidate for use in myeloma treatment (Lin, Owens, Tricot, & Wilson, 2004; Santonocito et al., 2004; Kim, Park, Medeiros, & Weissman, 2012; Hosen, 2013).

Daratumumab (Darzalex) is a first-in-class inhibitor of CD38 and the first monoclonal antibody approved for treatment of myeloma (Lokhorst et al., 2015). In November 2015, the US Food and Drug Administration (FDA) granted accelerated approval to daratumumab for the treatment of patients with myeloma who have received at least three prior lines of therapy, including a PI and an IMiD, or who are double-refractory to a PI and an IMiD. Further approval was granted by the FDA in November 2016 for the use of daratumumab in combination with 1) bortezomib and dexamethasone, or 2) lenalidomide and dexamethasone, for treatment of patients with multiple myeloma who have received at least one prior therapy.

## MECHANISM OF ACTION

Daratumumab is a human immunoglobulin (IgG1) monoclonal antibody directed against CD38, which is highly expressed on myeloma cells. It exerts antimyeloma activity through several mechanisms: (1) complement-dependent cytotoxicity (CDC); (2) antibody-dependent cell-mediated cytotoxicity (ADCC); (3) antibody-dependent cellular phagocytosis (ADCP); (4) enzymatic inhibition of CD38; and (5) direct induction of apoptosis upon secondary crosslinking. CD38 contributes to myeloma cell survival via adenosine production and subsequent calcium mobilization. Accordingly, inhibition of these functions is thought to contribute to the cytotoxic effect of daratumumab (de Weers et al., 2011; Overdijk et al., 2015).

Furthermore, daratumumab has been shown to induce immunomodulatory effects. CD38 is expressed on subsets of regulatory T cells, B cells, and monocytes, indicating these cells are sensitive to treatment with daratumumab. These CD38-positive subpopulations are highly immunosuppressive. By targeting and eliminating these cells, daratumumab removes a mechanism of immunosuppression and enables an antimyeloma response. Adaptive immune responses leading to increased T-cell expansion, activation, and clonality have been reported following treatment with daratumumab, indicating the drug’s immunomodulatory role (Krejcik et al., 2016; Moreau et al., 2016).

## CLINICAL STUDIES

**SIRIUS**

Accelerated approval of daratumumab was based upon the multicenter, open-label, phase II SIRIUS trial, which enrolled 106 heavily pretreated patients with relapsed or refractory myeloma to receive daratumumab monotherapy at a dose of 16 mg/kg. Patients were eligible if they had received at least three prior lines of therapy, including a PI and an IMiD, or who were double-refractory to a PI and an IMiD. The primary endpoint was overall response rate (ORR), defined as a partial response (PR) plus a very good PR plus a complete response (CR) plus a stringent CR.

Responses were assessed using the International Myeloma Working Group (IMWG) criteria, which take into account changes in M-protein levels, as determined by serum protein electrophoresis (SPE) and immunofixation electrophoresis (IFE), the percentage of bone marrow plasma cells, and free light chain (FLC) ratios. By using the IMWG response criteria, the SIRIUS investigators provided a basis for evaluating daratumumab that is consistent with practice standards at many clinical centers. Secondary endpoints included duration of response, PFS, OS, and clinical benefit rate (minimal response plus ORR).

The overall response rate demonstrated in the SIRIUS trial was 29.2% (95% confidence interval [CI]: 21%–39%). Median time to first response was 1 month (range, 0.9–5.6 months). Median duration of response was 7.4 months (range, 5.5 months to not estimable [NE]), and median OS was 17.5 months (range, 13.7 months to NE). Of patients who responded to daratumumab, 25.8% had responses that deepened over time (Lonial et al., 2016).

**CASTOR**

Although the SIRIUS trial evaluated daratumumab in heavily pretreated patients, strong interest exists in using daratumumab earlier in relapsed myeloma. The randomized, controlled, open-label phase III CASTOR trial enrolled 498 patients who had received one or more prior lines of therapy to receive a regimen of bortezomib (Velcade) and dexamethasone either alone or in combination with daratumumab. Drug dosing as used in CASTOR is described in Table 1. Patients were excluded if their disease was refractory to bortezomib or another PI.

**Table 1:**
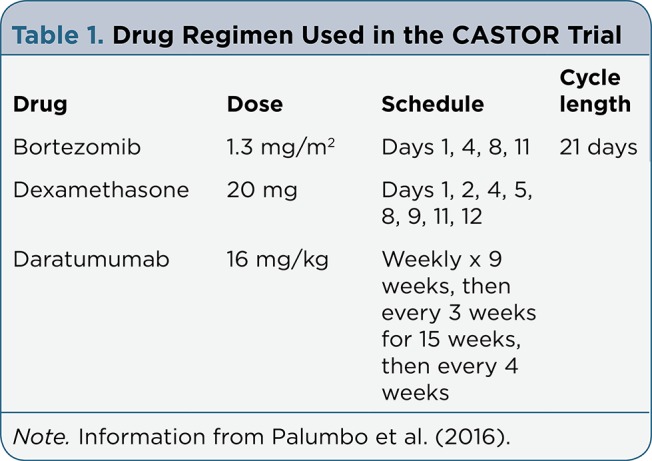
Drug Regimen Used in the CASTOR Trial

The primary endpoint was PFS, defined as the time from randomization to disease progression or death, whichever happened first. Responses were evaluated using the IMWG criteria. In patients who had a potential CR but daratumumab was suspected to have interfered with either SPE or IFE, additional reflex testing using an anti-idiotype antibody was performed to confirm the CR.

The CASTOR trial was halted due to a prespecified interim analysis showing significantly improved outcomes in the daratumumab group compared with the control group. Twelve-month PFS was 60.7% for daratumumab (95% CI: 51.2%–69%) vs. 26.9% for the control group (95% CI: 17.1%–37.5%). Median PFS was not reached at the time of interim analysis for the daratumumab group but was 7.2 months in the control group (95% CI: 6.2–7.9 monthas). The hazard ratio for disease progression or death for the daratumumab group vs. the control group was 0.39 (95% CI: 0.28–0.53, p < .001), representing a 61.4% lower risk of disease progression or death with the daratumumab group.

Other outcomes from the CASTOR trial can be found in Table 2. Overall, daratumumab was associated with higher rates of neutropenia (17.7% vs. 9.3%) and thrombocytopenia (58.8% vs. 43.9%) than the control group, as well as infusion-related reactions (45.3%). Patients in the daratumumab group with deeper responses (very good PR or better) were noted to have a greater benefit in PFS than those in the control group, although median PFS had not been reached at the time of interim analysis. Additional IFE reflex testing was performed to account for daratumumab interference on SPE. No very good PRs were reclassified as either a CR or a stringent CR as a result of the reflex IFE—an interesting finding on daratumumab interference on SPE that bears further study. Although the results of the CASTOR trial are encouraging, the full effects of daratumumab in combination with bortezomib and dexamethasone are pending accrual of longer-term follow-up data (Palumbo et al., 2016).

**Table 2:**
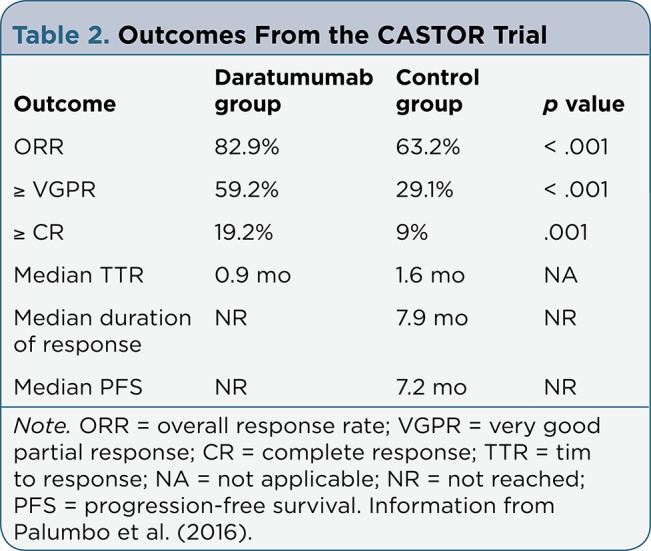
Outcomes From the CASTOR Trial

**POLLUX**

In Greek mythology, Castor and Pollux were twins who helped Jason and the Argonauts in their quest for the Golden Fleece, making it only natural that a companion trial to CASTOR be named POLLUX. The POLLUX trial was a randomized, controlled, open-label, phase III study that enrolled 569 patients who had received one or more prior lines of therapy to receive lenalidomide (Revlimid) and dexamethasone either alone or in combination with daratumumab. Drug dosing used in the POLLUX trial is described in Table 3. Patients were excluded if they had disease refractory to lenalidomide or had previously discontinued lenalidomide secondary to adverse events. The study’s primary endpoint was PFS, and responses were assessed using the IMWG criteria. Due to potential inference from daratumumab on SPE and IFE, patients with a suspected CR were evaluated using a daratumumab-specific IFE reflex assay.

**Table 3:**
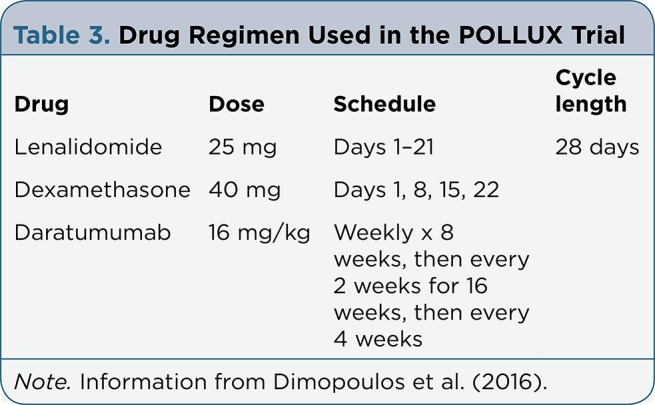
Drug Regimen Used in the POLLUX Trial

As with the CASTOR trial, the POLLUX trial was halted early due to a protocol-specified interim analysis. Twelve-month PFS was 83.2% in the daratumumab group (95% CI: 78.3%–87.2%) and 60.1% (95% CI: 54.0%–65.7%) in the control group. Median PFS was not reached in the daratumumab group but was 18.4 months (95% CI: 13.9 months to NE) in the control group. The hazard ratio for disease progression or death in the daratumumab group vs. the control group was 0.37 (95% CI: 0.27–0.52, p < .001), representing a 63% lower risk of disease progression or death in the daratumumab group.

Other notable outcomes from the POLLUX study can be found in Table 4. Overall, daratumumab was associated with a higher rate of neutropenia (51.9% vs. 37.0%) than the control group, as well as infusion-related reactions (47.7%). Although responses were first reported in the daratumumab group at 1 month, several months of treatment were required for a CR.

**Table 4:**
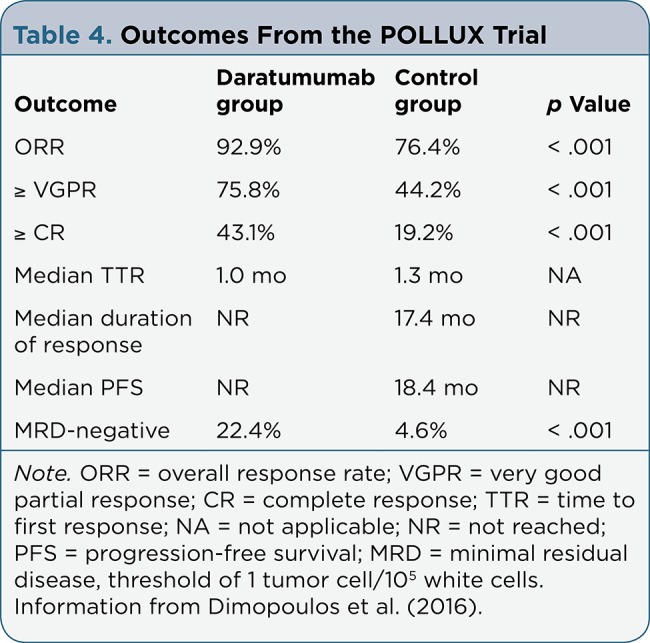
Outcomes From the POLLUX Trial

Consistent with outcomes from the CASTOR trial, patients in the POLLUX trial who received daratumumab displayed increased rates of deeper responses, with deeper responses resulting in longer PFS intervals. These results may be explained in part by the immunomodulatory synergy between daratumumab and lenalidomide, as described in preclinical studies (van der Veer et al., 2011b). In addition, the POLLUX trial demonstrated that daratumumab increased PFS regardless of the number of prior treatments—a finding that may impact when and where daratumumab is ultimately used in myeloma therapy. With only 12-month follow-up data reported thus far from the POLLUX trial, outcomes from longer-term follow-up are highly anticipated (Dimopoulos et al., 2016).

## DOSING AND ADMINISTRATION

Daratumumab is dosed at 16 mg/kg of body weight and given via intravenous (IV) infusion weekly for weeks 1 to 8, every 2 weeks for weeks 9 to 24, and every 4 weeks from week 25 until disease progression. Due to the risk of infusion-related reactions, daratumumab should be administered using recommended dilution volumes, infusion rates, and both pre-and postinfusion prophylaxis (Table 5). Preinfusion prophylaxis should include a corticosteroid, antipyretic, and antihistamine, and postinfusion prophylaxis should include a corticosteroid. Many centers incorporate a leukotriene receptor antagonist to further reduce the risk of an infusion reaction (Moreau et al., 2016). Dose adjustments of daratumumab are not necessary for preexisting renal impairment or mild hepatic impairment (total bilirubin 1–1.5 times the upper limit of normal [ULN] or aspartate transaminase greater than the ULN; Janssen Biotech, 2015).

**Table 5:**
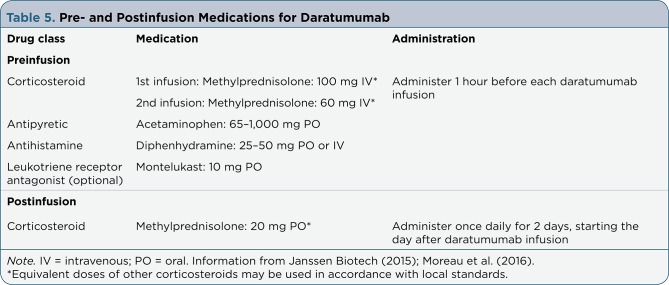
Pre- and Postinfusion Medications for Daratumumab

## SAFETY PROFILE AND ADVERSE EFFECTS

Adverse effects from daratumumab are largely characterized as grade 1 or 2, with fatigue (39%) and nausea (27%) as prevalent nonhematologic events. With CD38 expressed by both lymphoid and myeloid cell lines, hematologic adverse effects are common with daratumumab, including all-grade anemia (45%), neutropenia (60%), thrombocytopenia (48%), and lymphopenia (72%). Due to the underlying immunodeficiency of myeloma and preexisting cytopenias in many patients, the hematologic effects of daratumumab necessitate careful monitoring. Daratumumab administration in vivo is associated with an approximate decrease in hemoglobin of 1.6 mg/dL, with a compensatory increase in reticulocytes. The decrease in hemoglobin is considered to be a result of ADCP clearance of daratumumab-tagged red blood cells in the spleen, rather than CDC (Oostendorp et al., 2015; Moreau et al., 2016).

The major nonhematologic adverse effect with daratumumab is an infusion reaction, with 48% of patients experiencing a reaction of any grade with the first infusion. In the SIRIUS trial, the incidence of infusion reaction decreased to 5% with the second infusion and 4% with subsequent infusions. No reactions of grade 3 or higher were observed during second or subsequent treatments (Lonial et al., 2016; Janssen Biotech, 2015).

More recent data from the CASTOR and POLLUX trials confirm these numbers, as infusion reactions were noted in 45.3% of CASTOR patients receiving daratumumab, with 98.2% of reactions developing with the first dose. Similarly, 47.7% of patients receiving daratumumab in the POLLUX trial developed an infusion reaction, with 92% of reactions occurring with the first dose. The median time to onset of an infusion reaction is 1.5 hours, with nearly all reactions occurring during the infusion or within 4 hours of its completion. Infusion reactions are possible for up to 48 hours following infusion of daratumumab (Janssen Biotech, 2015).

## CURRENT PLACE IN THERAPY

Both clinical studies and the FDA approval status of daratumumab support its use in the setting of relapsed and refractory myeloma. Currently available data are remarkable, as daratumumab monotherapy produced an OS benefit that was better than expected when compared with historical data (Kumar et al., 2014). Furthermore, daratumumab monotherapy in relapsed and refractory myeloma resulted in a greater ORR (29.2%) than reported for other available therapies in the same setting, including bortezomib (27%), lenalidomide (26%), carfilzomib (24%), and pomalidomide (18%; Richardson et al., 2003, 2009, 2014; Siegel et al., 2012).

Although outcomes from daratumumab monotherapy are encouraging, the greatest benefit of daratumumab appears to be when it is used in combination with standard-of-care regimens, as seen in the CASTOR and POLLUX trials. The results from the CASTOR trial demonstrate the advantages of combination therapy with daratumumab and bortezomib, which may stem from the enhanced direct cytotoxicity noted against myeloma cells during in vitro preclinical studies when antibodies targeting CD38 were combined with PIs (van der Veer et al., 2011a). Similarly, the immunomodulatory synergy between daratumumab and lenalidomide as described in the POLLUX trial provides the clinical basis for combination therapy with other IMiDs.

However, currently available clinical trials have been unable to define daratumumab’s ultimate place in therapy. Data indicating increased rates of deeper responses, responses that deepen over time, and results below the threshold of minimal residual disease (MRD) suggest a role for daratumumab in the front-line setting. However, numerous combinations of active agents are possible, raising questions regarding the number of drugs needed to provide optimal results. Perhaps a combination of daratumumab, an IMiD, a PI, and dexamethasone may yield the best outcomes, but which IMiD? Which PI? What dose of dexamethasone? What are the nature and duration of maintenance therapy? The prevailing sentiment in the oncology community suggests that daratumumab may revolutionize myeloma treatment—just as rituximab (Rituxan) did for lymphoma—but much work remains to be done.

Extensive clinical trials to evaluate daratumumab in combination with other myeloma therapies are ongoing in both the frontline and relapsed/refractory settings. Other intriguing studies are examining the role of all-trans retinoic acid (ATRA) to up-modulate CD38 receptor density, providing more targets for daratumumab—a concept initially reported by Chillemi and colleagues (Chillemi et al., 2013). Neither the exact place in therapy nor the optimal treatment strategy for daratumumab is known yet, but we make progress toward these goals each day.

At the time of this writing, the National Comprehensive Cancer Network (NCCN) Guidelines for multiple myeloma support the use of daratumumab for patients with previously treated myeloma (NCCN, 2016).

## IMPLICATIONS FOR ADVANCED PRACTITIONERS

Daratumumab offers the advanced practitioner a new option for treatment of relapsed and refractory myeloma, principally in patients who have received at least three prior lines of therapy, including a PI and an IMiD. Due to the current unmet need in this area, daratumumab is likely to be used frequently.

**Infusion Reactions**

As discussed previously, daratumumab carries a risk of infusion reactions. This risk is mitigated by the use of prophylactic pre- and postinfusion medications, as well as larger dilutions and slower infusion rates during the initial doses. Although a combination of an antipyretic, antihistamine, and a corticosteroid is recommended for prevention of infusion reactions, leukotriene receptor antagonists such as montelukast are often added in practice. Emerging data indicate that giving montelukast prior to the first dose of daratumumab reduced the rate of infusion reactions by one-third, with patients experiencing fewer respiratory and gastrointestinal symptoms.

As reported in the CASTOR and POLLUX trials, common symptoms of infusion reactions are dyspnea, cough, and bronchospasm, with nasal congestion and rhinitis/rhinorrhea possible as well. These respiratory symptoms are likely due to CD38 expression by smooth muscle cells in the airways, making the addition of montelukast as a premedication an intriguing yet potentially useful option (Chari et al., 2016).

The POLLUX researchers used split dosing of dexamethasone, so patients’ therapeutic doses served as both treatment as well as pre- and postinfusion prophylaxis—a strategy that may be useful for advanced practitioners. For example, if a patient was receiving dexamethasone 40 mg weekly as part of the regimen, 20 mg would be administered prior to daratumumab as infusion reaction prophylaxis, and 20 mg would be administered the following day (Dimopoulos et al., 2016).

**Transfusion Concerns**

Several other considerations for the advanced practitioner exist with the use of daratumumab. Daratumumab binds to CD38 on red blood cells, causing panreactivity in vitro (Chapuy et al., 2015). This results in a positive indirect antiglobulin test and interferes with antibody screening and cross-matching, causing both safety concerns and delays in issuing units of blood for transfusions. Positive indirect antiglobulin tests may persist for up to 6 months following the last dose of daratumumab (Oostendorp et al., 2015). However, daratumumab does not interfere with the determination of a patient’s ABO and Rh blood type.

Resolving daratumumab’s interference with blood compatibility testing requires specific blood bank methods that are tailored to each institution. One method of mitigating daratumumab interference with red blood cell screening is to treat reagent red blood cells with dithiothreitol (DTT), which disrupts the extracellular bonds of CD38 and prevents daratumumab from binding to red blood cells. Other methods of neutralizing daratumumab include using soluble recombinant human CD38 or anti-idiotype antibodies, but these approaches are not yet widely available (American Association of Blood Banks, 2016).

Advanced practitioners can employ several strategies to minimize transfusion issues for their patients receiving daratumumab. A baseline type and screen should be performed prior to initiating daratumumab, ideally alongside a baseline phenotype. Additionally, patients should be provided with a transfusion identification card noting that (1) they are receiving daratumumab and (2) their blood profile (ABO type, Rh, and indirect antiglobulin test results) as determined prior to starting daratumumab. This identification card would be carried throughout the duration of therapy and for 6 months after discontinuation of daratumumab (Oostendorp et al., 2015; Moreau et al., 2016).

**Interference in Monitoring**

Daratumumab is an IgG monoclonal antibody and has been detected on SPE and IFE assays. When SPE and IFE are used to monitor endogenous monoclonal immunoglobulins (M-proteins) in IgG kappa myeloma patients treated with daratumumab, false-positive results are possible. The presence of daratumumab on SPE and IFE may thus interfere with the determination and validation of both CRs and very good PRs. This can be especially problematic in patients with anM-spike of less than 2 g/L, as daratumumab reaches peak serum concentrations of approximately 1 g/L at the end of the weekly dosing period—making it difficult to determine what portion of the M-spike is due to daratumumab and what portion is due to remaining M-protein (Moreau et al., 2016; McCudden et al., 2016). However, a daratumumab-specific immunofixation electrophoresis assay that can distinguish between the M-protein and the monoclonal antibody is available, and may help to remedy the issue of daratumumab interference (McCudden et al., 2016).

**Herpes Zoster Reactivation**

Reactivation of the herpes zoster virus is possible with daratumumab. A total of 73% of patients in clinical trials leading to daratumumab’s initial FDA approval used systemic antiviral agents; antiviral prophylaxis is recommended within 1 week of starting daratumumab and for 3 months following treatment.

**Cost Issues**

Finally, cost is a concern with daratumumab. Although the actual cost varies from institution to institution due to contract pricing, the average wholesale price (AWP) for a 400-mg vial of daratumumab was approximately $2,160 at the time of this writing (UpToDate, 2016). It is important to note that several vials would be needed to achieve a full dose for most patients, and that the cost listed here represents only daratumumab, which is increasingly likely to be used as part of a doublet or triplet regimen. Published costs for the triplet regimen of bortezomib, lenalidomide, and dexamethasone are in excess of $150,000/person/year, and the addition of daratumumab poses an even greater financial burden (Kantarjian & Rajkumar, 2015).

In general terms, the AWP for daratumumab is approximately 25% less than that of elotuzumab (Empliciti), a monoclonal antibody targeting SLAMF7 that is currently approved in the same setting. However, when the total cost of regimens including daratumumab is considered, this is but a Pyrrhic victory.

## SUMMARY

The introduction of monoclonal antibodies is likely to shift the paradigm of myeloma treatment. The efficacy and safety demonstrated to date by first-in-class daratumumab give practitioners a new option for treatment of relapsed or refractory myeloma, either as monotherapy or in combination with available agents.

The combination of efficacy, response rate in heavily pretreated patients, and favorable safety profile make daratumumab an especially exciting option for patients with myeloma. Data from early-phase trials as well as from the CASTOR and POLLUX trials demonstrate that daratumumab can be safely incorporated to existing backbone regimens—resulting in increased efficacy and response rates (Plesner et al., 2014; Moreau et al., 2014; Dimopoulos et al., 2016; Palumbo et al., 2016). As studies progress, we can expect to see daratumumab approved for use in combination with available agents, added to currently active backbone regimens, and ultimately moved to the front-line setting in myeloma treatment.
